# Supplementary material for: Hierarchical modelling of factors associated with the practice and perpetuation of female genital mutilation in the next generation of women in Africa
Source: PLoS One. 2021 Apr 23;16(4):e0250411. doi: 10.1371/journal.pone.0250411 (PMC8064566; doi:10.1371/journal.pone.0250411)
Supplement: S1 Table — (DOCX) [file pone.0250411.s001.docx]

S1 Table: Distribution of the sampled women, proportion with living children, proportion asked question on daughters’ circumcision and valid responses by country

| Country | Total sample size of women | Number with living daughters | % with living daughters | Number asked daughter FGM | % asked daughter FGM | % with valid response | Number with valid response |
| --- | --- | --- | --- | --- | --- | --- | --- |
| Nigeria | 41,821 | 24,464 | 58.5 | 8,725 | 35.7 | 8291 | 95.0 |
| Kenya | 31,079 | 18,554 | 59.7 | 7,548 | 40.7 | 7226 | 95.7 |
| Tanzania | 13,266 | 7,704 | 58.1 | 5,983 | 77.7 | 5912 | 98.8 |
| Senegal | 16,787 | 8,931 | 53.2 | 8,013 | 89.7 | 7684 | 95.9 |
| Ethiopia | 15,683 | 8,198 | 52.3 | 3,906 | 47.6 | 3829 | 98.0 |
| Burkina Faso | 17,087 | 10,732 | 62.8 | 10,156 | 94.6 | 10064 | 99.1 |
| Guinea | 10,874 | 6,192 | 56.9 | 5,606 | 90.5 | 5559 | 99.2 |
| Cote d’Ivoire | 10,060 | 5,892 | 58.6 | 4,965 | 84.3 | 4864 | 98.0 |
| Mali | 10,519 | 6,628 | 63.0 | 3,165 | 47.8 | 3147 | 99.4 |
| Niger | 11,160 | 6932 | 62.1 | 3505 | 50.6 | 3244 | 92.6 |
| Sierra Leone | 16,658 | 9892 | 59.4 | 9781 | 98.9 | 9759 | 99.8 |
| Togo | 9,480 | 5,512 | 58.1 | 3,751 | 68.1 | 3561 | 94.9 |
| Chad | 17,719 | 11,937 | 67.4 | 5,906 | 49.5 | 5724 | 96.9 |
| Egypt | 21,762 | 15,323 | 70.4 | 14,497 | 94.6 | 14198 | 97.9 |
|  |  |  |  |  |  |  |  |
| Total | 243,955 | 146,891 | 60.2 | 95,507 | 65.0 | 93,063 | 97.8 |
