# Supplementary material for: Hierarchical modelling of factors associated with the practice and perpetuation of female genital mutilation in the next generation of women in Africa
Source: PLoS One. 2021 Apr 23;16(4):e0250411. doi: 10.1371/journal.pone.0250411 (PMC8064566; doi:10.1371/journal.pone.0250411)
Supplement: S2 Table — (DOCX) [file pone.0250411.s002.docx]

S2 Table: Comparison of mothers’ and daughters’ age at circumcision

| Age {x) | Daughter at Age x | Mother at Age x | Daughter Cumulative | Mother Cumulative |
| --- | --- | --- | --- | --- |
| 0 | 36.9 | 29.8 | 36.9 | 29.8 |
| 1 | 7.8 | 0.8 | 44.7 | 30.6 |
| 2 | 8.5 | 1.3 | 53.2 | 31.9 |
| 3 | 6.6 | 1.5 | 59.8 | 33.4 |
| 4 | 4.8 | 2.0 | 64.6 | 35.4 |
| 5 | 7.1 | 4.6 | 71.7 | 40.0 |
| 6 | 5.3 | 5.0 | 77.0 | 45.0 |
| 7 | 5.5 | 6.2 | 82.5 | 51.1 |
| 8 | 3.3 | 6.4 | 85.8 | 57.5 |
| 9 | 2.7 | 5.8 | 88.5 | 63.3 |
| 10 | 4.7 | 13.5 | 93.2 | 76.8 |
| 11 | 2.0 | 3.9 | 95.1 | 80.7 |
| 12 | 2.5 | 7.0 | 97.6 | 87.7 |
| 13 | 0.7 | 2.4 | 98.3 | 90.0 |
| 14 | 0.3 | 1.7 | 98.6 | 91.7 |
| 15 | 0.2 | 2.4 | 98.7 | 94.0 |
| 16 | 0.0 | 0.8 | 98.8 | 94.8 |
| 17 | 0.0 | 0.4 | 98.8 | 95.2 |
| 18 | 0.0 | 0.4 | 98.8 | 95.6 |
| 19 | 0.0 | 0.1 | 98.8 | 95.7 |
| 20+ | 0.2 | 0.5 | 99.0 | 96.2 |
| DK | 1.0 | 3.8 | 100.0 | 100.0 |
