# Supplementary material for: Hierarchical modelling of factors associated with the practice and perpetuation of female genital mutilation in the next generation of women in Africa
Source: PLoS One. 2021 Apr 23;16(4):e0250411. doi: 10.1371/journal.pone.0250411 (PMC8064566; doi:10.1371/journal.pone.0250411)
Supplement: Attachment — Submitted filename: Plos One revisions.docx [file pone.0250411.s003.docx]

September 13^th^ 2020

Dear Editor

PLOS ONE

Re: PONE-D-20-19500
Perpetration of female genital mutilation in the next generation of women from 12 African countries: Evidence from hierarchical modelling of their attributes

We the authors of above mentioned paper appreciate the efforts and comments of the editor and the eminent reviewers. We have addressed all these comments. A point-by-point response to the issues in our revised manuscript is listed below.

Please note that we used the file with the tracked changes to describe where the changes were made.

Editor Comments:

Journal Requirements:

When submitting your revision, we need you to address these additional requirements.

1. Please ensure that your manuscript meets PLOS ONE's style requirements, including those for file naming. The PLOS ONE style templates can be found at

https://journals.plos.org/plosone/s/file?id=wjVg/PLOSOne_formatting_sample_main_body.pdf and

https://journals.plos.org/plosone/s/file?id=ba62/PLOSOne_formatting_sample_title_authors_affiliations.pdf

 THANK YOU. WE HAVE FORMATTED THE ENTIRE MANUSCRIPT TO MEET PLOS ONE STYLES

2. We suggest you thoroughly copyedit your manuscript for language usage, spelling, and grammar. If you do not know anyone who can help you do this, you may wish to consider employing a professional scientific editing service.  

Whilst you may use any professional scientific editing service of your choice, PLOS has partnered with both American Journal Experts (AJE) and Editage to provide discounted services to PLOS authors. Both organizations have experience helping authors meet PLOS guidelines and can provide language editing, translation, manuscript formatting, and figure formatting to ensure your manuscript meets our submission guidelines. To take advantage of our partnership with AJE, visit the AJE website (http://learn.aje.com/plos/) for a 15% discount off AJE services. To take advantage of our partnership with Editage, visit the Editage website (www.editage.com) and enter referral code PLOSEDIT for a 15% discount off Editage services.  If the PLOS editorial team finds any language issues in text that either AJE or Editage has edited, the service provider will re-edit the text for free.

 Upon resubmission, please provide the following:

- The name of the colleague or the details of the professional service that edited your manuscript
- A copy of your manuscript showing your changes by either highlighting them or using track changes (uploaded as a *supporting information* file)
- A clean copy of the edited manuscript (uploaded as the new *manuscript* file)

 THANK YOU. WE HAVE RNGAGED THE SERVICES OF NATIVE ENGLISH EDITOR IN THE COPYEDITTING

3. We note that Figures 1 and 2 in your submission contain map images which may be copyrighted.

All PLOS content is published under the Creative Commons Attribution License (CC BY 4.0), which means that the manuscript, images, and Supporting Information files will be freely available online, and any third party is permitted to access, download, copy, distribute, and use these materials in any way, even commercially, with proper attribution. For these reasons, we cannot publish previously copyrighted maps or satellite images created using proprietary data, such as Google software (Google Maps, Street View, and Earth). For more information, see our copyright guidelines: http://journals.plos.org/plosone/s/licenses-and-copyright.

 We require you to either (a) present written permission from the copyright holder to publish these figures specifically under the CC BY 4.0 license, or (b) remove the figures from your submission:

 a. You may seek permission from the original copyright holder of Figures 1 and 2 to publish the content specifically under the CC BY 4.0 license.

 We recommend that you contact the original copyright holder with the Content Permission Form (http://journals.plos.org/plosone/s/file?id=7c09/content-permission-form.pdf) and the following text:

“I request permission for the open-access journal PLOS ONE to publish XXX under the Creative Commons Attribution License (CCAL) CC BY 4.0 (http://creativecommons.org/licenses/by/4.0/). Please be aware that this license allows unrestricted use and distribution, even commercially, by third parties. Please reply and provide explicit written permission to publish XXX under a CC BY license and complete the attached form.”

 Please upload the completed Content Permission Form or other proof of granted permissions as an "Other" file with your submission.

 In the figure caption of the copyrighted figure, please include the following text: “Reprinted from [ref] under a CC BY license, with permission from [name of publisher], original copyright [original copyright year].”

 b. If you are unable to obtain permission from the original copyright holder to publish these figures under the CC BY 4.0 license or if the copyright holder’s requirements are incompatible with the CC BY 4.0 license, please either i) remove the figure or ii) supply a replacement figure that complies with the CC BY 4.0 license. Please check copyright information on all replacement figures and update the figure caption with source information. If applicable, please specify in the figure caption text when a figure is similar but not identical to the original image and is therefore for illustrative purposes only.

The following resources for replacing copyrighted map figures may be helpful:

 USGS National Map Viewer (public domain): http://viewer.nationalmap.gov/viewer/

The Gateway to Astronaut Photography of Earth (public domain): http://eol.jsc.nasa.gov/sseop/clickmap/

Maps at the CIA (public domain): https://www.cia.gov/library/publications/the-world-factbook/index.html and https://www.cia.gov/library/publications/cia-maps-publications/index.html

NASA Earth Observatory (public domain): http://earthobservatory.nasa.gov/

Landsat: http://landsat.visibleearth.nasa.gov/

USGS EROS (Earth Resources Observatory and Science (EROS) Center) (public domain): http://eros.usgs.gov/#

Natural Earth (public domain): http://www.naturalearthdata.com/

THE GEOGRAPHICAL MAPS AND OBJECTS USED IN THE FIGURES WERE NOT COPYRIGHTED. HOWEVER, WE HAVE OBTAINED A WRITTEN PERMISSION FROM THE OWNER (SEE THE ATTACHMENT IN THE “OTHERS”). THE TWO FIGURES WERE DRAWN BY THE AUTHORS, THE TEXTS IN THE FIGURES ARE THE WORKS OF THE AUTHORS.

4. We note that Figure 1 includes images of individuals.

As per the PLOS ONE policy (http://journals.plos.org/plosone/s/submission-guidelines#loc-human-subjects-research) on papers that include identifying, or potentially identifying, information, the individual(s) or parent(s)/guardian(s) must be informed of the terms of the PLOS open-access (CC-BY) license and provide specific permission for publication of these details under the terms of this license.

Please download the Consent Form for Publication in a PLOS Journal (http://journals.plos.org/plosone/s/file?id=8ce6/plos-consent-form-english.pdf).

The signed consent form should not be submitted with the manuscript, but should be securely filed in the individual's case notes. Please amend the methods section and ethics statement of the manuscript to explicitly state that the patient/participant has provided consent for publication: “The individual in this manuscript has given written informed consent (as outlined in PLOS consent form) to publish these case details”.

If you are unable to obtain consent from the subject of the photograph, you will need to remove the figure and any other textual identifying information or case descriptions for these individuals.

 THANK YOU. WE HAVE REPLACED THE HUMAN IMAGES WITH NON-COPYRIGHTED OBJECTS

5. Please ensure that language is neutral and factual throughout the manuscript. We would for instance ask that you consider whether the term 'perpetration' in the title can be changed to a more neutral term.

 THANK YOU. WE AGREE WITH YOU AND CONSIDERED “PROCUREMENT PERPETUATION” TO BE MORE NEUTRAL THAN PERPETRATION

6. Please amend either the abstract on the online submission form (via Edit Submission) or the abstract in the manuscript so that they are identical.

THANK YOU. THE ABSTRACTS ARE NOW THESAME

7. Please ensure that you refer to Figure 4 in your text as, if accepted, production will need this reference to link the reader to the figure.

FIGURE 4 HAVE BEEN CITED

8. Please include captions for your Supporting Information files at the end of your manuscript, and update any in-text citations to match accordingly. Please see our Supporting Information guidelines for more information: http://journals.plos.org/plosone/s/supporting-information

THANK YOU. WE HAVE PROVIDED THE CAPTIONS FOR THE SUPPORTING INFORMATION FILES AT THE END OF YOUR MANUSCRIPT…….
[Note: HTML markup is below. Please do not edit.]

Reviewers' comments:

Reviewer's Responses to Questions

**Comments to the Author**

1. Is the manuscript technically sound, and do the data support the conclusions?

The manuscript must describe a technically sound piece of scientific research with data that supports the conclusions. Experiments must have been conducted rigorously, with appropriate controls, replication, and sample sizes. The conclusions must be drawn appropriately based on the data presented.

Reviewer #1: Yes

Reviewer #2: Yes

 2. Has the statistical analysis been performed appropriately and rigorously?

 Reviewer #1: I Don't Know

Reviewer #2: Yes

 3. Have the authors made all data underlying the findings in their manuscript fully available?

The [PLOS Data policy](http://www.plosone.org/static/policies.action#sharing) requires authors to make all data underlying the findings described in their manuscript fully available without restriction, with rare exception (please refer to the Data Availability Statement in the manuscript PDF file). The data should be provided as part of the manuscript or its supporting information, or deposited to a public repository. For example, in addition to summary statistics, the data points behind means, medians and variance measures should be available. If there are restrictions on publicly sharing data—e.g. participant privacy or use of data from a third party—those must be specified.

 Reviewer #1: Yes

Reviewer #2: Yes

 4. Is the manuscript presented in an intelligible fashion and written in standard English?

PLOS ONE does not copyedit accepted manuscripts, so the language in submitted articles must be clear, correct, and unambiguous. Any typographical or grammatical errors should be corrected at revision, so please note any specific errors here.

 Reviewer #1: Yes

Reviewer #2: Yes

 5. Review Comments to the Author

Please use the space provided to explain your answers to the questions above. You may also include additional comments for the author, including concerns about dual publication, research ethics, or publication ethics. (Please upload your review as an attachment if it exceeds 20,000 characters)

**Reviewer #1:** I found the manuscript well written, informative and touches on a critical problem bewildering most of the developing countries. Though it is academic paper, it will really benefit policy /decision makers who are willing to act on this historical injustice to women and girls.

THANK YOU, WE HOPE TO DEVELOP A POLICY BRIEF FROM THIS ACADEMIC PAPER

However, in section 2.3, line 119, the author mentioned that they will collect information or seek to understand on "how the FMG was done". maybe the author can recheck as it doesn't come out clearly in the results section.

THANK YOU, THIS WAS AN ERROR, CHECKING HOW FGM WERE DONE WAS OUTSIDE THE SCOPE OF THIS STUDY. WE HAVE REMOVED THE PHRASE.

On the other note, it would be interesting to understand why Catholics were looked at separately from other Christians. There seems to be no mention the role of Catholics in literature review.

WE SEPARATED THE CATHOLICS FROM THE OTHER CHRISTIANS BECAUSE LITERATURE SUGGESTED THEY HAVE SLIGHTLY DIFFERENT DOCTRINES ON SOME SEXUAL AND REPRODUCTIVE ISSUES

**Reviewer #2:** This is a well-carried out study that seeks to fill an important gap in reproductive literature in Africa. The paper was well written from background to conclusion.

THANK YOU

However, I have some few methodological suggestions which can help strengthen the paper.
1. Under line 114, the authors state “In this study, we analyzed data of 80403 girls from 7993 communities nested within 12 countries”. The authors should be clear whether the unit of analysis is ‘women with daughters’ or ‘girls’.

THANK YOU, THE UNIT OF ANALYSIS IS “WOMEN-DAUGHTER PAIRS”. WE HAVE CORRECTED THIS AND ELSEWHERE IN THE MANUSCRIPT

2. Under line 167-168, the authors state “Data were weighted and statistical significance determined at 5%”. Can the authors indicate how data was weighted?

THE DHS DATA PROVIDED SAMPLING WEIGHTS FOR EACH PARTCIAPNT. WE APPLIED THE SAMPLING WEIGHTS IN THE ANALYSIS. THE PROCESS OF APPLYING THE WEIGHT IS OFTEN REFERRED TO AS WEIGHTING, SO THE DATA WERE WEIGHTED. WE HAVE CHANGED THE STATEMENT TO READ “WE APPLIED SAMPLING WEIGHTS TO THE DATA”

3. Can the authors indicate how they accounted for the complex sampling design of the surveys?

FIRSTLY WE USED INTRA-COUNTRY WEIGHTING WHICH HAD ALREADY ACCOUNTED FOR THE COMPLEXITY IN TERMES OF UNEQUAL POPULATION SIZES IN EACH CLUSTERS, REGIONS AND STATES. MORE IMPORTANTLY, WE USED CLUSTERED ANALYSIS THROUH MULTI-LEVEL MODELLING THAT ACCOUNTED FOR THE HIERACHICAL NATURE OF THE DATA
4. The authors pooled data from 12 African countries. Where is weighting? I do not mean the DHS weighting, but I mean your data weighting after pooling as you deal with multiple countries with wide variation in their population. You can refer to the reference below to understand what I mean.
Marriott, B. M., Campbell, L., Hirsch, E., & Wilson, D. (2007). Preliminary data from demographic and health surveys on infant feeding in 20 developing countries. The Journal of nutrition, 137(2), 518S-523S.

WE APPRECIATE THIS IMPORTANT ISSUE RAISED BY OUR REVIEWER. WE DID NOT ADJUST FOR THE DIFFERENCES IN SAMPLE SIZES OF EACH COUNTRY FOR THE FOLLOWING REASONS

a. MOST STATISTICAL SOFTWARE DO NOT MAKE PROVISION FOR INTER-COUNTRY WEIGHTING, RATHER WEIGHTING IS AVAILABLE FOR INTRA-COUNTRY WEIGHTING
b. WEIGHTING, EITHER INTER OR INTRA-COUNTRY, DOES NOT AFFECT MULTIVARIATE ANALYSIS EXCEPT FREQUENCIES
c. THE GOAL OF THE STUDY WAS NOT TO ESTIMATE AN AFRICAN-WIDE FIGURES AS 12 COUNTRIES CANNOT POSSIBLY REPRESENT OVER 50 COUNTRIES, SO WE WERE MORE CONCERNED ABOUT COUNTRY-SPECIFIC ANALYSIS FOR ALL THE UNIVARIATE AND BIVARIATE ANALYSIS WE PRESENTED.
d. MARRIOTT ET AL ADJUSTED FOR VARIABILITY IN THE NUMBER OF INDIVIDUALS SAMPLED IN EACH COUNTRY AND NOT FOR POPULATION SIZES OF EACH COUNTRY.
e. MOST MULTI-COUNTRY STUDY DO NOT

5. Under line 170-172, the authors state “Respondents with no female child and respondents with no information on whether the daughter was circumcised or not were excluded from further analysis.” This is an indication that there was missing data in each country’s dataset, can the authors provide the following information for each country
A. total women interviewed
B. Sample size by design
C. Selected women sample (this is already in Table 1).
D. Percentage with complete information on the inclusion criteria
E. Percentage of missing data.

THANK YOU. THIS STATEMENT DID NOT CONSTITUTE MISSING DATA. IT WILL BE MISLEADING TO CLASSIFY ALL SAMPLED WOMEN WITH NO RESPONSE TO DAUGHTERS CIRCUMCISION AS MISSING. FIRSTLY THERE ARE NO WAYS WOMEN WITH NO DAUGHTER CAN PROVIDE INFORMATION ON DAUGHTERS CIRCUMCISION OR OTHERWISE, SECONDLY, GOING BY THE SAMPLING DESIGN, WOMEN WITH NO LIVING DAUGHTER WERE AUTOMATICALLY EXCLUDED. THIRDLY, WOMEN FROM HOUSEHOLDS THAT WERE SELECTED FOR “MAN INTERVIEW” WERE EXCLUDED FROM “FEMALE GENITAL MUTILATION” MODULE. WE HAD REFERED HAVE TO THE MOTHERS IN THE SECOND AND THIRD CATEGORIES AS THOSE “RESPONDENTS WITH NO INFORMATION ON WHETHER THE DAUGHTER WAS CIRCUMCISED OR NOT”.

THE SENTENCE HAVE BEEN REFRAMED TO READ “RESPONDENTS WHO DID NOT GIVE BIRTH TO ANY FEMALE CHILD, OR WHO HAS NO LIVING DAUGHTER WERE WAS DEAD OR WHO WERE SELECTED FOR MALE QUESTIONNAIRE ACCORDING TO THE SURVEY PROTOCOL WERE NOT ASKED QUESTIONS ON DAUGHTER’S CIRCUMCISION. OF THE 81,921 ASKED QUESTIONS ON DAUGHTER’S CIRCUMCISION, 80,403(98.1%) PROVIDED VALID RESPONSES (SUPPLEMENTARY TABLE B). THE REMAINING 1.9% WERE EXCLUDED FROM FURTHER ANALYSIS.”

DETAILS OF THE METHODOLOGY IS AVAILABLE AT <https://dhsprogram.com/what-we-do/survey/survey-display-528.cfm>

6. Finally, can the authors explain how they treated missing data?

ACCORDING TO DHS, THE SAMPLING WEIGHTS ARE COMPUTED AFTER DATA COLLECTION, SO THE SAMPLING WEIGHTS HAVE ACCOUNTED FOR NON RESPONSES AND UNEQUAL POPULATION SIZES IN EACH GEOGRAPHICAL DOMAINS. (<https://dhsprogram.com/data/Guide-to-DHS-Statistics/index.htm#t=Analyzing_DHS_Data.htm>). THE 1.9% INVALID RESPONSES WERE TREATED AS MISSING

 6. PLOS authors have the option to publish the peer review history of their article ([what does this mean?](https://journals.plos.org/plosone/s/editorial-and-peer-review-process#loc-peer-review-history)). If published, this will include your full peer review and any attached files.

If you choose “no”, your identity will remain anonymous but your review may still be made public.

**Do you want your identity to be public for this peer review?** For information about this choice, including consent withdrawal, please see our [Privacy Policy](https://www.plos.org/privacy-policy).

 Reviewer #1: **Yes:** Patrick O. Okoth

Reviewer #2: **Yes:** Bright Opoku Ahinkorah

 [NOTE: If reviewer comments were submitted as an attachment file, they will be attached to this email and accessible via the submission site. Please log into your account, locate the manuscript record, and check for the action link "View Attachments". If this link does not appear, there are no attachment files.]

While revising your submission, please upload your figure files to the Preflight Analysis and Conversion Engine (PACE) digital diagnostic tool, <https://pacev2.apexcovantage.com/>. PACE helps ensure that figures meet PLOS requirements. To use PACE, you must first register as a user. Registration is free. Then, login and navigate to the UPLOAD tab, where you will find detailed instructions on how to use the tool. If you encounter any issues or have any questions when using PACE, please email PLOS at [figures@plos.org](mailto:figures@plos.org). Please note that Supporting Information files do not need this step.

 WE HAVE VERIFIED THE FIGURES AND UPLOADED ACCORDINGLY

Yours truly

ADENIYI FAGBAMIGBE
