# Supplementary material for: Hierarchical modelling of factors associated with the practice and perpetuation of female genital mutilation in the next generation of women in Africa
Source: PLoS One. 2021 Apr 23;16(4):e0250411. doi: 10.1371/journal.pone.0250411 (PMC8064566; doi:10.1371/journal.pone.0250411)
Supplement: Attachment — Submitted filename: Plos One revisions R2.docx [file pone.0250411.s004.docx]

November 23^rd^ 2020

Dear Editor

PLOS ONE

Re: PONE-D-20-19500R1
Perpetration of female genital mutilation in the next generation of women from 12 African countries: Evidence from hierarchical modelling of their attributes

We the authors of above mentioned paper appreciate the efforts and comments of the editor and the eminent reviewers. We have addressed all these comments. A point-by-point response to the issues in our revised manuscript is listed below.

Please note that we used the file with the tracked changes to describe where the changes were made.

PONE-D-20-19500R1
Hierarchical modelling of factors associated with the practice and perpetuation of female genital mutilation in the next generation of women in Africa
PLOS ONE

Dear Dr. Fagbamigbe,

Thank you for submitting your manuscript to PLOS ONE. After careful consideration, we feel that it has merit but does not fully meet PLOS ONE’s publication criteria as it currently stands. Therefore, we invite you to submit a revised version of the manuscript that addresses the points raised during the review process.

Please submit your revised manuscript by Jan 04 2021 11:59PM. If you will need more time than this to complete your revisions, please reply to this message or contact the journal office at plosone@plos.org. When you're ready to submit your revision, log on to https://www.editorialmanager.com/pone/ and select the 'Submissions Needing Revision' folder to locate your manuscript file.

Please include the following items when submitting your revised manuscript:

- A rebuttal letter that responds to each point raised by the academic editor and reviewer(s). You should upload this letter as a separate file labeled 'Response to Reviewers'.
- A marked-up copy of your manuscript that highlights changes made to the original version. You should upload this as a separate file labeled 'Revised Manuscript with Track Changes'.
- An unmarked version of your revised paper without tracked changes. You should upload this as a separate file labeled 'Manuscript'.

If you would like to make changes to your financial disclosure, please include your updated statement in your cover letter. Guidelines for resubmitting your figure files are available below the reviewer comments at the end of this letter.

1. If the authors have adequately addressed your comments raised in a previous round of review and you feel that this manuscript is now acceptable for publication, you may indicate that here to bypass the “Comments to the Author” section, enter your conflict of interest statement in the “Confidential to Editor” section, and submit your "Accept" recommendation.

Reviewer #2: All comments have been addressed

Thank you

2. Is the manuscript technically sound, and do the data support the conclusions?

The manuscript must describe a technically sound piece of scientific research with data that supports the conclusions. Experiments must have been conducted rigorously, with appropriate controls, replication, and sample sizes. The conclusions must be drawn appropriately based on the data presented.

Reviewer #2: Yes

Thank you

3. Has the statistical analysis been performed appropriately and rigorously?

Reviewer #2: Yes

Thank you

4. Have the authors made all data underlying the findings in their manuscript fully available?

The [PLOS Data policy](http://www.plosone.org/static/policies.action#sharing) requires authors to make all data underlying the findings described in their manuscript fully available without restriction, with rare exception (please refer to the Data Availability Statement in the manuscript PDF file). The data should be provided as part of the manuscript or its supporting information, or deposited to a public repository. For example, in addition to summary statistics, the data points behind means, medians and variance measures should be available. If there are restrictions on publicly sharing data—e.g. participant privacy or use of data from a third party—those must be specified.

Reviewer #2: Yes

Thank you

5. Is the manuscript presented in an intelligible fashion and written in standard English?

PLOS ONE does not copyedit accepted manuscripts, so the language in submitted articles must be clear, correct, and unambiguous. Any typographical or grammatical errors should be corrected at revision, so please note any specific errors here.

Reviewer #2: Yes

Thank you

6. Review Comments to the Author

Please use the space provided to explain your answers to the questions above. You may also include additional comments for the author, including concerns about dual publication, research ethics, or publication ethics. (Please upload your review as an attachment if it exceeds 20,000 characters)

Reviewer #2: I appreciate the efforts the authors have made in revising their manuscript per the comments of the editor and reviewers. I think all the issues I raised have been addressed. Although I consider the reasons the authors gave for not applying a weighting factor in the pooled data as not convincing, I accept their reasons in line with scholarly agreement.

Going through the revised paper, I have identified a statement which the authors made and which is not correct and needs to be addressed:
In line 98-99, the authors claimed “We identified only 12 countries with data set on FGM for both the respondents and their daughters.” This statement is not correct. This is because Sierra Leone is also a country in Africa with data set on FGM for both mother and daughter. In terms of prevalence of FGM in Africa, it is one of the countries with the highest prevalence of FGM (88%) (https://www.who.int/reproductivehealth/topics/fgm/prevalence/en/). I suggest the inclusion of Sierra Leone in this study as there seems to be no reason for leaving the country out.

Thank you. We totally agree with your comments. We had excluded Sierra Leone and Niger because it the dataset for these two countries did not capture some relevant information.

We have now included the two countries, reanalysed the data entirely and updated all relevant sections of the paper. The inclusion of these countries will definitely strengthen our findings.

7. PLOS authors have the option to publish the peer review history of their article ([what does this mean?](https://journals.plos.org/plosone/s/editorial-and-peer-review-process#loc-peer-review-history)). If published, this will include your full peer review and any attached files.


If you choose “no”, your identity will remain anonymous but your review may still be made public.


**Do you want your identity to be public for this peer review?** For information about this choice, including consent withdrawal, please see our [Privacy Policy](https://www.plos.org/privacy-policy).

Reviewer #2: **Yes:** Bright Opoku Ahinkorah

Thank you
