# Supplementary material for: Hierarchical modelling of factors associated with the practice and perpetuation of female genital mutilation in the next generation of women in Africa
Source: PLoS One. 2021 Apr 23;16(4):e0250411. doi: 10.1371/journal.pone.0250411 (PMC8064566; doi:10.1371/journal.pone.0250411)
Supplement: Attachment — Submitted filename: Plos One revisions R3.docx [file pone.0250411.s005.docx]

March 18^th^ 2021

Dear Editor

PLOS ONE

PONE-D-20-19500R2
Hierarchical modelling of factors associated with the practice and perpetuation of female genital mutilation in the next generation of women in Africa
PLOS ONE

We the authors of above mentioned paper appreciate the efforts and comments of the editor and the eminent reviewers. We have addressed all these comments. A point-by-point response to the issues in our revised manuscript is listed below.

Please note that we used the file with the tracked changes to describe where the changes were made.

PONE-D-20-19500R1
Hierarchical modelling of factors associated with the practice and perpetuation of female genital mutilation in the next generation of women in Africa
PLOS ONE

Dear Dr. Fagbamigbe,

Thank you for submitting your manuscript to PLOS ONE. After careful consideration, we feel that it has merit but does not fully meet PLOS ONE’s publication criteria as it currently stands. Therefore, we invite you to submit a revised version of the manuscript that addresses the points raised during the review process.

Please submit your revised manuscript by Jan 04 2021 11:59PM. If you will need more time than this to complete your revisions, please reply to this message or contact the journal office at plosone@plos.org. When you're ready to submit your revision, log on to https://www.editorialmanager.com/pone/ and select the 'Submissions Needing Revision' folder to locate your manuscript file.

Please include the following items when submitting your revised manuscript:

- A rebuttal letter that responds to each point raised by the academic editor and reviewer(s). You should upload this letter as a separate file labeled 'Response to Reviewers'.
- A marked-up copy of your manuscript that highlights changes made to the original version. You should upload this as a separate file labeled 'Revised Manuscript with Track Changes'.
- An unmarked version of your revised paper without tracked changes. You should upload this as a separate file labeled 'Manuscript'.

If you would like to make changes to your financial disclosure, please include your updated statement in your cover letter. Guidelines for resubmitting your figure files are available below the reviewer comments at the end of this letter.

Thank you. We have addressed all these

**Comments to the Author**

1. If the authors have adequately addressed your comments raised in a previous round of review and you feel that this manuscript is now acceptable for publication, you may indicate that here to bypass the “Comments to the Author” section, enter your conflict of interest statement in the “Confidential to Editor” section, and submit your "Accept" recommendation.

Reviewer #2: All comments have been addressed

Reviewer #3: (No Response)

Thank you

2. Is the manuscript technically sound, and do the data support the conclusions?

The manuscript must describe a technically sound piece of scientific research with data that supports the conclusions. Experiments must have been conducted rigorously, with appropriate controls, replication, and sample sizes. The conclusions must be drawn appropriately based on the data presented.

Reviewer #2: Yes

Reviewer #3: Yes

Thank you

3. Has the statistical analysis been performed appropriately and rigorously?

Reviewer #2: Yes

Reviewer #3: Yes

Thank you

4. Have the authors made all data underlying the findings in their manuscript fully available?

The [PLOS Data policy](http://www.plosone.org/static/policies.action#sharing) requires authors to make all data underlying the findings described in their manuscript fully available without restriction, with rare exception (please refer to the Data Availability Statement in the manuscript PDF file). The data should be provided as part of the manuscript or its supporting information, or deposited to a public repository. For example, in addition to summary statistics, the data points behind means, medians and variance measures should be available. If there are restrictions on publicly sharing data—e.g. participant privacy or use of data from a third party—those must be specified.

Reviewer #2: Yes

Reviewer #3: Yes

Thank you

5. Is the manuscript presented in an intelligible fashion and written in standard English?

PLOS ONE does not copyedit accepted manuscripts, so the language in submitted articles must be clear, correct, and unambiguous. Any typographical or grammatical errors should be corrected at revision, so please note any specific errors here.

Reviewer #2: Yes

Reviewer #3: Yes

Thank you

6. Review Comments to the Author

Please use the space provided to explain your answers to the questions above. You may also include additional comments for the author, including concerns about dual publication, research ethics, or publication ethics. (Please upload your review as an attachment if it exceeds 20,000 characters)

Reviewer #2: Thank you for spending time to incorporate the suggestions. All comments have been addressed. Well done.

Thank you

Reviewer #3: A research study was conducted to access the current practice of mother and daughter FGM (female genital mutilation). A hierarchical multivariable logistic regression model was fitted. The results indicate that the risk of mothers having FGM for their daughters was significantly associated with the following factors: maternal age, educational status, religion, household wealth quintiles, place of residence, community unemployment and community poverty.

Minor revisions:
1- Lines 135 and 192: Improve the clarity of the statement by revising these sentences.

Thank you. We have revised the sentences to read “We included only respondents (women) with response on at least one daughter’s FGM status in this study. This was to allow mother-daughter analysis of FGM practice. Our analysis may, therefore, differ slightly from the published estimates on level of FGM among women and children by the DHS for each of the countries.”

and

“The remaining 2.2% with invalid responses on FGM among daughters were excluded from further analysis.” respectively

2- Line 270: Revise “mean mothers’ age” to “mothers’ mean age.”
Thank you

3- P-values never equal zero; express small p-values as p < 0.001.
Thank you. We have changed all the appearances

4- Table 4: to conform to standard practice, provide only the overall p-value for each factor rather than p-values for each level of each factor.

Thank you. We appreciate your comment and would have been idea if the p-value is from a chi-square. But this is not the case here, we carried out a logistic regression, where all estimate for each levels were compared with the reference level for each factor. It is possible that one level is significant while the other is not significant. For example, marital status in Fig 4: never married people had significant lower odds than the currently married people, but the formerly married people had insignificant odds compared with the currently married people. It will therefore be wrong to use a single p-value for the two. Therefore, the current presentation of p-values are correct.

7. PLOS authors have the option to publish the peer review history of their article ([what does this mean?](https://journals.plos.org/plosone/s/editorial-and-peer-review-process#loc-peer-review-history)). If published, this will include your full peer review and any attached files.


If you choose “no”, your identity will remain anonymous but your review may still be made public.


**Do you want your identity to be public for this peer review?** For information about this choice, including consent withdrawal, please see our [Privacy Policy](https://www.plos.org/privacy-policy).

Reviewer #2: **Yes:** Bright Opoku Ahinkorah

Reviewer #3: No

 Thank you
